# Derivation of the Difficult Airway Physiological Score (DAPS) in adults undergoing endotracheal intubation in the emergency department

**DOI:** 10.1186/s12873-024-00958-3

**Published:** 2024-03-12

**Authors:** Shahan Waheed, Junaid Abdul Razzak, Nadeemullah Khan, Ahmed Raheem, Asad Iqbal Mian

**Affiliations:** 1 Department of Emergency Medicine, Aga Khan University & Hospital (AKUH), Karachi, Pakistan; 2 Department of Emergency Medicine, New York Presbyterian Weill Cornell Medicine, New York, USA

**Keywords:** Emergency medicine, Difficult airway, Physiological airway, Prediction, Endotracheal intubation

## Abstract

**Background:**

Prediction of serious outcomes among patients with physiological instability is crucial in airway management. In this study, we aim to develop a score to predict serious outcomes following intubation in critically ill adults with physiological instability by using clinical and laboratory parameters collected prior to intubation.

**Method:**

This single-center analytical cross-sectional study was conducted in the Emergency Department from 2016 to 2020. The airway score was derived using the transparent reporting of a multivariable prediction model for individual prognosis or diagnosis (TRIPOD) methodology. To gauge model’s performance, the train-test split technique was utilized. The discrete random number generation approach was used to divide the dataset into two groups: development (training) and validation (testing). The validation dataset’s instances were used to calculate the final score, and its validity was measured using ROC analysis and area under the curve (AUC). By computing the Youden’s J statistic using the metrics sensitivity, specificity, positive predictive value, and negative predictive value, the discriminating factor of the additive score was determined.

**Results:**

The mean age of the 1021 patients who needed endotracheal intubations was 52.2 years (± 17.5), and 632 (62%) of them were male. In the development dataset, there were 527 (64.9%) physiologically difficult airways, 298 (36.7%) post-intubation hypotension, 124 (12%) cardiac arrest, 347 (42.7%) shock index > 0.9, and 456 [56.2%] instances of pH < 7.3. On the contrary, in the validation dataset, there were 143 (68.4%) physiologically difficult airways, 33 (15.8%) post-intubation hypotension, 41 (19.6%) cardiac arrest, 87 (41.6%) shock index > 0.9, and 121 (57.9%) had pH < 7.3, respectively. There were 12 variables in the difficult airway physiological score (DAPS), and a DAPS of 9 had an area under the curve of 0.857. The accuracy of DAPS was 77%, the sensitivity was 74%, the specificity was 83.3%, and the positive predictive value was 91%.

**Conclusion:**

DAPS demonstrated strong discriminating ability for anticipating physiologically challenging airways. The proposed model may be helpful in the clinical setting for screening patients who are at high risk of deterioration.

**Supplementary Information:**

The online version contains supplementary material available at 10.1186/s12873-024-00958-3.

## Introduction

Critically ill patients with physiological instability presenting to the emergency room encounter many challenges. Controlling the airway is the most crucial skill in these situations since it can prevent serious outcomes [1]. The procedure has the potential to save the patient’s life if an accurate and prompt evaluation is made. Difficult airways occur between 0.4% and 8.5% in anesthesia practice, but between 2 and 14% in an emergency department practice [2]. Data is lacking regarding emergency department intubations and the management of difficult airways from developing countries. There is no general definition of the term “difficult airway,” and its usage varies significantly across literature [3–5]. The emergency medical literature frequently examines both anatomical and physiological aspects when discussing difficult airways [1, 6]. The four dimensions of difficult airway are difficult bag mask ventilation, difficult glottic device use, difficult laryngoscopy, and difficult cricothyroidotomy [7]. Physiologically challenging airways are those in which patient with severe physiological abnormalities enhance the risk of cardiovascular collapse and mortality after intubation or during the transition to positive pressure breathing [8].

Difficult airway prediction scores in anesthesia and intensive care units are frequently used to evaluate the entire spectrum of difficult airway [7, 9]. Despite the widely held idea that physiological instability takes primacy in emergency departments. Most of these assessments place a focus on anatomical criteria while giving physiological factors little to no importance. The prognosis of patients could be significantly improved with the early diagnosis of specific physiological abnormalities and related treatment. Hypoxia, hypotension, severe metabolic acidosis, and right ventricular failure are believed to all contribute to physiologically difficult airways. In the chaotic environment of an emergency, however, these ratings present a major obstacle. Detecting right ventricular failure using echocardiography, for example, requires skills that vary among physicians. Further, availability of ultrasound machines in emergency departments varies across the country which prevents their use and incorporation in difficult airway management guidelines. There are two scores (the HEAVEN criteria [10] and the MACOCHA scores [11]) that utilize few physiological markers from emergency medical literature. The shock index, pH, and presentation systolic blood pressure were not included in the evaluations of cardiovascular and metabolic parameters in these scores. It is also difficult to identify instances in which these ratings could be useful in emergency care.

Therefore, the objective of this research is to create a difficult airway physiological score (DAPS) for critically ill adults with physiological instability undergoing endotracheal intubation in the emergency department by analyzing pre-intubation clinical and laboratory information.

## Methods

### Study design & site

This cross-sectional, single-center study was conducted in the emergency department of a large urban academic hospital with 62 beds and 60,000 annual patient visits. The TRIPOD statement served as the basis for the diagnostic prediction method used to derive the score [12].

### Subjects and definition of a physiologic difficult airway

The study cohort consists of ≥ 18-year-old adults who required emergency department endotracheal intubation throughout a five-year period (January 2016–December 2020). Patients brought in after cardiac arrest, when airway management may be difficult owing to continuous CPR, patients with oropharyngeal tumors with deformed airway anatomy, and pregnant women with different physiological abnormalities were precluded from treatment. In our study, we defined physiologic difficult airways as having any one of the following characteristics in patients presenting to the emergency room and needing endotracheal intubation: hypoxia, defined as oxygen saturation less than 92%; hypotension, defined as systolic blood pressure less than 90 mmHg or hypotension that is resistant to fluid resuscitation; metabolic acidosis, defined as pH less than 7.3; and shock index ≥ 0.9.

### Data collection

All patients who had endotracheal intubation in the emergency department during the previous five years (2016–2020) were collected from our electronic health records, utilizing the International Categorization of Diseases-10 (ICD–10) as the disease classification system. Prior to official data collection, a pilot test was conducted on the data collection questionnaire. ICD-10 is a medical coding system that was primarily developed by the World Health Organization (WHO) to classify health issues by illness categories under which more specific conditions are mentioned. The data was obtained using paper questionnaires that were piloted on 10 samples prior to the commencement of the formal data collection. The pilot samples were excluded from the study’s final analysis. The data was collected by research assistants who were trained to collect emergency department airway management variables since they have worked on many emergency airway management studies in the past. Every day, an emergency physician evaluates the data collection form to check for missing data and discrepancies. On the proforma, data variables include demographic information, clinical presentation, history and concomitant conditions, laboratory data, physician notes, vital monitoring data, and discharge narratives. REDCap electronic data capture tool maintained by Aga Khan University was used to gather and manage study data. REDCap (Research Electronic Data Capture) is a secure, web-based software platform designed to facilitate the collection of data for research investigations [13]. This study was conducted considering the principles of ethics under the “Declaration of Helsinki.” [14].

### Serious outcomes

The serious outcomes were the composite of primary and secondary outcomes. The primary outcomes were hypotension (defined as a reduction in systolic blood pressure < 90 mmHg pressure or hypotension that is resistant to fluids only and necessitates the addition of vasopressor therapy following endotracheal intubation) and hypoxia (defined as the inability to oxygenate with oxygen saturations < 92% post-intubation). Cardiac arrest (defined as the absence of a pulse during or after endotracheal intubation) and mortality (defined as death occurring within 1 h after intubation in the emergency department) were the secondary endpoints.

### Data analysis

All statistical analyses were conducted using version 22.0 of IBM SPSS Statistics for Windows (Armonk, New York, IBM Corp.). A comprehensive collection of demographics, clinical, laboratory, and outcome data was obtained for the study. Even though the approach of mean imputation was utilized for some laboratory assessment data with 5% missing observations, such as ABGs, the analysis was ignored. On the entire dataset, descriptive analysis was conducted, and the association between worsening hypotension/worsening hypoxia/cardiac arrest/mortality and various demographic, clinical, and laboratory characteristics was evaluated using the Chi square/Fisher’s test or independent sample t-test/Mann-Whitney U test, as appropriate. Continuous variables such as age (years), time when endotracheal intubation was performed, systolic and diastolic blood pressures (mmHg), heart rate (beats/min), pH, oxygen saturations (%), and shock index were dichotomized according to clinically defined criteria for the receiver operating characteristics (ROC) curve analysis. This produces a total of 12 variables that were identified as potential indicators of serious outcomes (post-intubation hypotension, worsening hypoxemia, cardiac arrest, and mortality). These features were then used to develop a predictive model. We did not include two variables (succinylcholine and rocuronium), as these were not present at the time of decision-making for endotracheal intubation in the emergency department. Since we use the medications after we make the decision to intubate, they are not included in our final score.

Due to the absence of an independent validation cohort, the train-test split method was used to evaluate the performance of the model. Using the discrete random number generation method in Microsoft Excel 2020, the data set was divided into two groups: the development (training) and the validation (testing) dataset, with a 0.80 probability that every given occurrence would be assigned to the development dataset. 80% of the examples used in the design and development of the prediction model were chosen at random from the construction dataset. The remaining 20% of examples from the validation data set were holdouts and were not incorporated into the building of the model. Using these holdout cases, the validity and performance of the model was assessed by comparing the disparities in distribution between the two datasets. Using univariate logistic regression analysis, potential variables for the subsequent multivariable model generation approach were selected. The stepwise backward conditional selection method was used to conduct a multivariable binary logistic regression analysis, with probabilities of 0.05 and 0.10 for entry and exit, respectively. The odds ratio (OR) and 95% confidence interval (CI) were determined for the univariate, initial, and final solutions of the multivariable regression analysis model. The evaluation consisted of a categorization table (confusion matrix) and the Hosmer-Lemeshow test for assessments of goodness-of-fit. Based on the rounded ORs generated, the score points of the variables in the final model were evaluated, and an additive score was then calculated. Validity was determined by computing the ROC curve, area under the curve (AUC), and 95% confidence interval for the final score (CI). The major discriminating point of the additive score was established by computing Youden’s J statistic and the sensitivity, specificity, positive predictive value (PPV), and negative predictive value (NPV) of the score at many probable discriminating points. A p-value of 0.05 was considered statistically significant in all analyses.

## Results

The study consists of 1021 participants, with 634 (62%) male and 387 (38%) female having a mean age of 52.2 (+ 17.5) years. Most intubations 399 (39%) were performed at night (10 p.m.– 8 a.m.), with shortness of breath being the frequently observed presentation 45.2%, followed by drowsiness (GCS < 15) 38.9%. The dataset was split as per one or more of the characteristics of physiologically difficult airway (which includes; hypotension, hypoxia, metabolic acidosis and shock index ≥ 0.9). A total of 670 participants has physiologically difficult airway on presentation (Table 1).

**Table 1:** Emergency department intubation characteristics among patients as per physiological difficult airway (presentation hypotension, hypoxia, metabolic acidosis pH < 7.3 and shock index  ≥.9)

Characteristics	Total 1021	Physiological difficult airway		p-value
		Yes (670)	No (351)	
**Age (years)**	52.2 (± 17.5)	53.6 (± 17.2)	49.3 (± 17.7)	< 0.001*
**Age groups**				
< 45 Years	336 [32.9%]	189 [28.2%]	147 [41.9%]	< 0.001*
≥ 45 Years	685 [67.1%]	481 [71.8%]	204 [58.1%]	
**Gender**				
Male	634 [62.1%]	385 [57.5%]	249 [70.9%]	< 0.001*
Female	387 [37.9%]	285 [42.5%]	102 [29.1%]	
**Shifts**				
Morning (8AM-4PM)	330 [32.3%]	212 [31.6%]	118 [33.6%]	0.812
Evening (4PM-10PM)	292 [28.6%]	194 [29%]	98 [27.9%]	
Night (10PM-8AM)	399 [39.1%]	264 [39.4%]	135 [38.5%]	
**Pre intubation Vitals** **Median** [IQR]				
Systolic Blood pressure (mmHg)	130 (153 − 110)	125 (149 − 105)	136.5 (160 − 117)	< 0.001*
Diastolic Blood pressure (mmHg)	77 (90 − 62)	72 (88 − 60)	80 (92 − 70)	< 0.001*
Heart Rate	106 (122 − 88)	109 (124 − 90)	102 (120 − 84)	0.010*
Oxygen Saturations (%)	94 (98 − 81)	93 (98 − 79)	96 (98 − 88)	< 0.001*
Respiratory Rate	25 (32 − 20)	27 (32 − 20)	24 (30 − 20)	< 0.001*
**Reasons for intubation**				
Coma	39 [3.8%]	24 [3.6%]	15 [4.3%]	0.584
Hypoxia	119 [11.7%]	81 [12.1%]	38 [10.9%]	0.550
Metabolic Acidosis	136 [13.3%]	101 [15.1%]	35 [10%]	0.023*
Anticipated Decline	147 [14.4%]	111 [16.6%]	36 [10.3%]	0.006*
Respiratory Distress	687 [67.3%]	501 [74.8%]	186 [53%]	< 0.001*
Polytrauma	24 [2.4%]	13 [1.9%]	11 [3.1%]	0.232
Isolated trauma	32 [3.1%]	17 [2.5%]	15 [4.3%]	0.130
Gunshot Injury	10 [1%]	4 [0.6%]	6 [1.7%]	0.086
Others	29 [2.8%]	22 [3.3%]	7 [2%]	0.239
**Shock Index**				
< 0.9	587 [57.5%]	343 [51.2%]	244 [69.5%]	< 0.001*
≥ 0.9	434 [42.5%]	327 [48.8%]	107 [30.5%]	
**pH**				
≥ 7.3	444 [43.5%]	220 [32.8%]	224 [63.8%]	< 0.001*
< 7.3	577 [56.5%]	450 [67.2%]	127 [36.2%]	
**Induction agent**				
Ketamine	79 [7.7%]	61 [9.1%]	18 [5.1%]	0.024*
Propofol	306 [30.1%]	192 [28.8%]	114 [32.5%]	0.205
Etomidate	233 [22.8%]	164 [24.5%]	69 [19.7%]	0.081
Midazolam	492 [48.2%]	316 [47.2%]	176 [50.3%]	0.366
**Paralyzing agent**				
Succinylcholine	632 [61.9%]	385 [57.5%]	247 [70.4%]	< 0.001*
Rocuronium	79 [7.8%]	47 [7%]	32 [9.1%]	0.233
Atracurium	293 [28.7%]	224 [33.5%]	69 [19.7%]	< 0.001*
**HEAVEN Criteria**				
Hypoxemia	414 [40.5%]	309 [46.1%]	105 [29.9%]	< 0.001*
Extremes of Size	22 [2.2%]	13 [1.9%]	9 [2.6%]	0.514
Anatomic abnormalities	35 [3.4%]	24 [3.6%]	11 [3.1%]	0.709
Vomiting/Blood/Fluid	219 [21.4%]	183 [27.3%]	36 [10.3%]	< 0.001*
Exsanguination	17 [1.7%]	10 [1.5%]	7 [2%]	0.552
Neck Mobility issues	60 [5.9%]	47 [7%]	13 [3.7%]	0.033*

In-hospital mortality was statistically significant in 281 (70.6%) versus 117 [33.5%] for patients with or without physiological difficult airway, p-value 0.027. Death in emergency was observed in 7 individuals of which 6 [0.9%] had physiologic difficult airway versus 1(0.3%) with no physiologic abnormality, p = value 0.027. Likewise, post intubation hypotension was observed in 379 (OR 0.45 [0.42–0.49]) patients. Cardiac arrest was observed in 124 (OR 0.44 [0.35–0.58]), and oxygen saturation < 92% was observed in 131 (OR 0.61 [0.57–0.64]) patients with physiological difficult airway (Table 2).

**Table 2:** Characteristics of outcome of patients as per physiological difficult airway parameters

Outcome	Total 1021	Physiological difficult airway		p-value	Odds Ratio
		Yes (670)	No (351)		
Cardiac Arrest	124 [100%]	124 [100%]	0 [0%]	< 0.001*	0.44 [0.35–0.58]
Hypotension (SBP < 100 mmHg)	379 [100%]	379 [100%]	0 [0%]	< 0.001*	0.45 [0.42–0.49]
Oxygen Saturation (< 92%)	131 [100%]	131 [100%]	0 [0%]	< 0.001*	0.61 [0.57–0.64]
Death in Emergency (within 1 h after intubation)	7 [100%]	6 [85.7%]	1 [0.3%]	0.027*	
Death in Hospital	398 [100%]	281 [70.6%]	117 [33.5%]		
Discharge	492 [100%]	303 [61.6%]	189 [54.2%]		

A total of 812 (80%) participants were randomly assigned to the development dataset and 209 (20%) to the validation dataset. The physiologically difficult airway was observed in 527 (64.9%) in the development group versus 143 (68.4%) in the validation group. There was no statistical difference between the development and the validation group (Supplementary Table 1). In the modeling-fitting phase, 27 significantly independent predictors for physiologically difficult airways were identified. The identified variables were selected with adjusted OR (95% CI). The score points for each variable were assigned as per the OR (95% CI) and p-value (Table 3 and Supplementary Fig. 1).

**Table 3:** Multivariable binary logistic regression analysis and difficult airway physiological score schema of the 12 predictor variables of the score for the prediction of serious outcomes among patients with physiological difficult airway during endotracheal intubation

Parameters	Multivariable		Score points
	OR (95% CI)	P-value	
Female gender	1.49 [1.07–2.07]	0.017*	1
Age ≥ 45 years	1.58 [1.13–2.19]	0.007*	2
Shift Duty (Morning-Evening)	2.12 [1.55–2.9]	< 0.001*	2
Presentation Hypotension	1.99 [1.19–3.34]	0.009*	2
Presentation Respiratory Distress	1.68 [1.17–2.42]	0.005*	2
Vomiting	3.41 [2.17–5.35]	< 0.001*	3
Shock Index ≥ 0.9	1.68 [1.33–2.57]	0.032*	2
pH group < 7.3	4.39 [3.17–6.09]	< 0.001*	4
Fever	2.13 [1.38–3.3]	< 0.001*	2
Anticipated decline in unstable patients	1.89 [1.16–3.06]	0.01*	2
GCS < 15	1.66 [1.14–2.41]	0.008*	2
Agitation	1.42 [1 -2.02]	0.048*	1

Hosmer and Lemeshow Test (Chi-square = 11.619, degree of freedom (d.f) = 8, *p* = 0.169

**Fig. 1:**
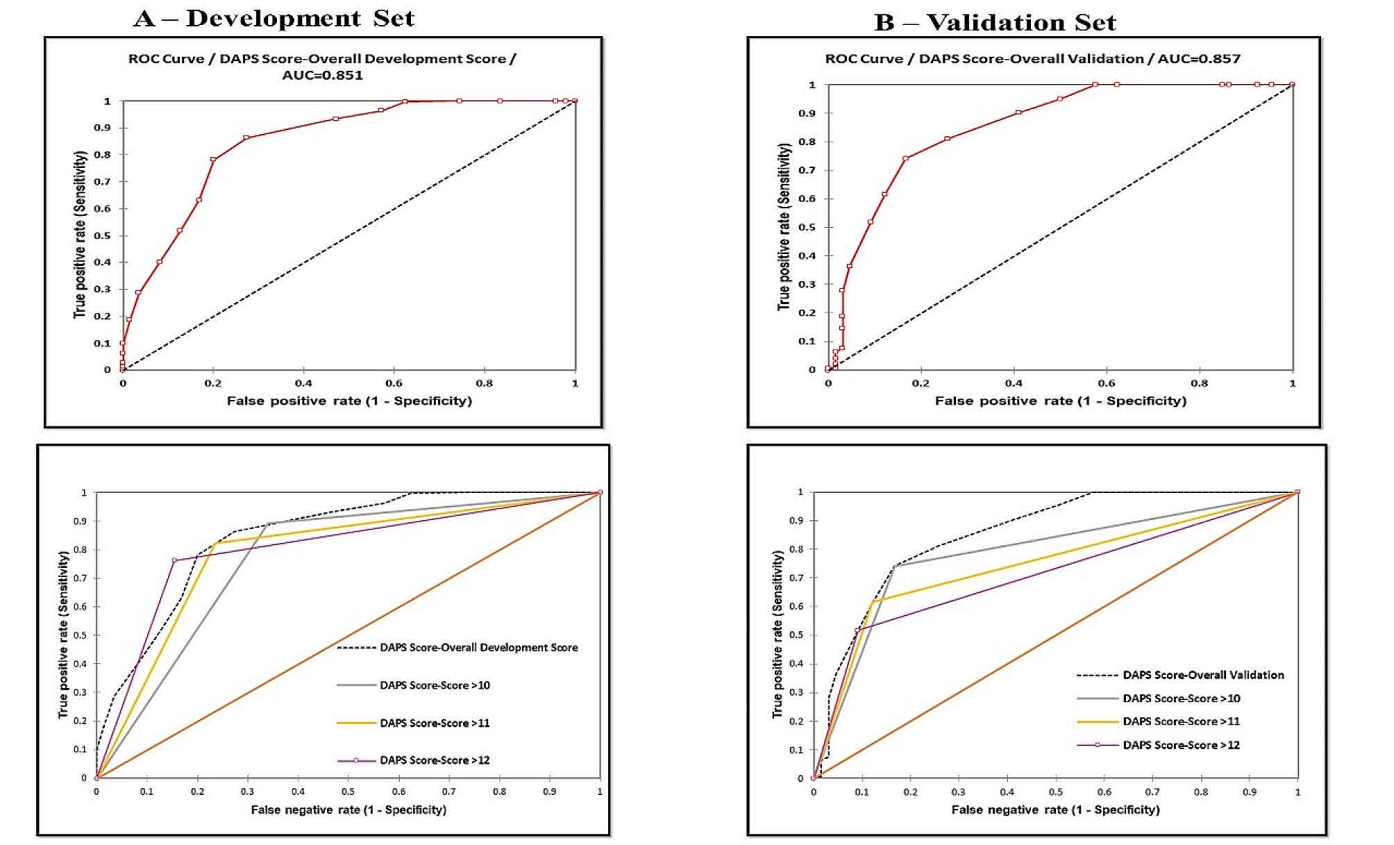
ROC curve analysis of the difficult airway physiological score (DAPS) in the development (**A**) and validation (**B**) data showing the overall score AUC and AUC based on score quartiles

**Fig. 2:**
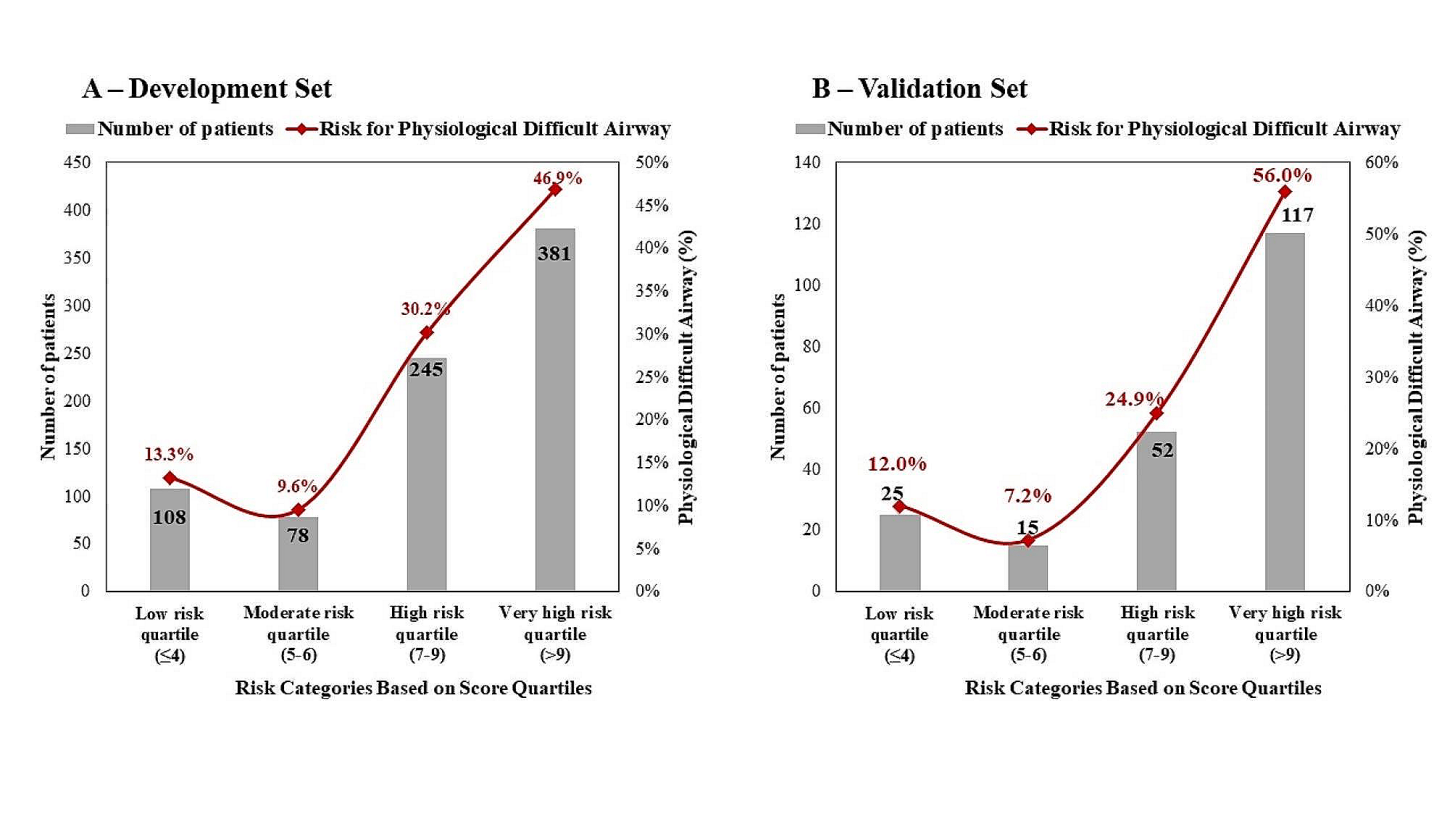
Risk stratification score for the difficult airway physiological score post endotracheal intubation in development (**A**) and validation (**B**) dataset

In the ROC curve analysis, the AUC of the derived score was found to be 0.851 (0.799–0.915) in the development and 0.857(0.823–0.887) in the validation group (Fig. 1). The 4 quartiles of the score were categorized as low-risk (≤ 4), moderate-risk (5–6), high-risk [7–9], and very high-risk (> 9), with a percentage of 13.3%, 9.6%, 30.2%, and 46.9%, respectively, in the development group. In the validation dataset, the quartiles were 12%, 7.2%, 24.9%, and 56%. (Fig. 2).

In the development dataset the optimal cut-off value for the DAPS at a score of ≥ 9, had a sensitivity of 81.8% [78.2–84.8%], specificity 73% [67.5–77.8%], PPV 84.84%, NPV 68.42%, and an accuracy of 78.69% (Supplementary Table 2). Whereas in the validation dataset, the optimal cut-off value for the DAPS at a score of ≥ 9, was found to have a sensitivity of 74.1% [66.3–80.6%], specificity 83.3% [72.3–90.5%], PPV 91%, NPV 60%, and an accuracy of 77% (Supplementary Table 3).

## Discussion

The difficult airway physiological score helps identify patients who presents with physiological instability in the emergency department and are at risk of serious outcomes (hypotension, desaturation, cardiac arrest, and mortality) following endotracheal intubation. It is incumbent to assess both anatomical and physiological predictors of difficulty simultaneously and early to prevent catastrophic outcome [15, 16].. Our score consists of 12 variables; Female gender, age ≥ 45 years, shift in which intubation occurs, presentation hypotension, presentation respiratory distress, vomiting, shock index ≥ 0.9, pH < 7.3, fever, anticipated decline due to unstable patient clinical condition, agitation, and GCS < 15. All these variables are included in our additive scoring system as predictors of serious outcomes. The objective of our score is to aid emergency physicians in making safe decisions by using pre-intubation clinical and laboratory information and averting post-intubation severe consequences.

Our study shows that pH < 7.3 is one of the biggest independent predictors of difficult physiological airway by contributing 4 points in our additive scoring model. Literature suggests that metabolic acidosis is one of the predictors of cardiovascular collapse post intubation [8, 17, 18]. In patients with severe metabolic acidosis, acid production demands on alveolar ventilation cannot be met, as seen in diabetic ketoacidosis (DKA), salicylate poisoning, or severe lactic acidosis. Additionally, during laryngoscopy there is a brief phase of apnea that further worsens the acidosis due to accumulation of carbon dioxide [8]. It is therefore necessary that non-invasive ventilation should be considered to prevent early intubation if airway control is not immediately needed [19].

In our score, vomiting is the second predictor variable that shares the highest score of 3. Vomiting is an extremely rare but deadly presentation in the emergency department that leads to severe hypoxemia and poor visualization of the vocal cords during laryngoscopy [10]. Soiled airways, a medical jargon indicating pulmonary aspiration of blood or vomitus, renders intubation or mask ventilation impossible. This is an extremely harmful situation, and the fallout might be catastrophic if measures aren’t taken quickly. Pulmonary aspiration in the elderly with poor functional status and physiological instability can make the situation much worse. It is an important variable to consider while managing airways and is included in our score. Two points each came from the two most significant cardiovascular predictors, presentation hypotension and shock index ≥ 0.9. Both factors are independent indicators of a physiologically difficult airway since they have been associated with hypotension both during and after maintenance of a definitive airway. Both emergency department and intensive care unit (ICU) literature examined the relationship between the pre-intubation shock index and subsequent hypotension and cardiac arrest [17, 20–23]. This leads to a consensus among ED studies by establishing the cut-off of shock index as predictor of poor outcomes at around 0.8 or higher [24]. The shock index may be easily evaluated in the ED for patients undergoing intubation; therefore, adding it to DAPS will enhance the screening process.

Age ≥ 45 years, morning/evening shift work, respiratory distress upon presentation, fever, anticipated decline in unstable patients as assessed by physician, and a GCS of < 15 have 2 points each. Female gender and agitation on presentation at the emergency department has a score of 1. Various factors contribute to the complexity of physiologically difficult airways [25].. Clinical and laboratory variables have been identified as key predictors of difficult physiological airway. After endotracheal intubation, patients who are hemodynamically stable have a 30% chance of experiencing severe cardiovascular collapse and 4% experiencing cardiac arrest [26], compared to a 1 in 10,000 likelihood reported in anesthesia literature [2, 7]. This effect can be due to drugs or underlying physiological instability. The difficult airway physiological score exhibits good accuracy and offers a reliable prediction of serious outcomes in a dynamic emergency department environment where severity and turnover of patient is rapid.

It is expected that the Difficult Airway Physiological Score will be useful in the clinical practice for predicting serious outcomes among patients with physiological instability in the emergency department. The DAPS score, a novel 12-variable risk stratification model, demonstrated high discriminatory power, high sensitivity, and high specificity in predicting an airway that would be physiologically challenging. It is possible that despite the suggested model having useful therapeutic application, further information is needed to validate the score externally.

### Limitations

There are certain limitations to this study. The primary concern is the lack of external validation, and the extent to which the suggested scoring system can be applied to different situations has yet to be determined through everyday testing. Data from a single site and retrospective nature prevented evaluation of some potentially significant prognostic markers such as time of presentation and subsequent intubation, resuscitation attempts prior to intubation, and rapid sequence intubation drug dosages. Secondly, the accuracy and consistency of the clinically recorded data impeded the use of historical data. We are hopeful, however, that this data will be included in emergency medicine’s standard of care for airway management. Third, there was no collection of important recognized indications of a physiologically difficult airway, such as right heart failure and metabolic acidosis; it is not standard practice to have a blood gas or an echo before endotracheal intubation.

It is essential to emphasize that the proposed score is intended for use in conjunction with other pertinent patient data in the context of life-threatening scenarios necessitating urgent airway intervention. Notwithstanding the identified limitations, the application of the Difficult Airway Physiological Score (DAPS) has the potential to contribute significantly to the domain of emergency medicine. Notably, the predictive capabilities of the DAPS offer valuable insights that may aid emergency physicians in enhancing their anticipatory assessment of patients with physiologically challenging airways, thus potentially fostering improved patient outcomes.

The outcomes of this study serve as a foundational platform, laying the groundwork for subsequent impact analyses aimed at gathering further empirical evidence. The potential modification of emergency physicians’ clinical approaches in the assessment of patients with physiological instability, facilitated by the integration of the DAPS, represents a compelling area for future research and clinical implementation.

### Electronic supplementary material

Below is the link to the electronic supplementary material.

Supplementary Material 1

## Data Availability

The datasets produced and/or examined during the present investigation are not accessible to the public to uphold participant confidentiality regulations. However, they can be obtained from the corresponding author upon a reasonable request.
