# Supplementary material for: Derivation of the Difficult Airway Physiological Score (DAPS) in adults undergoing endotracheal intubation in the emergency department
Source: BMC Emerg Med. 2024 Mar 12;24:40. doi: 10.1186/s12873-024-00958-3 (PMC10929237; doi:10.1186/s12873-024-00958-3)
Supplement: Supplementary file 1 — Supplementary Material 1 [file 12873_2024_958_MOESM1_ESM.docx]

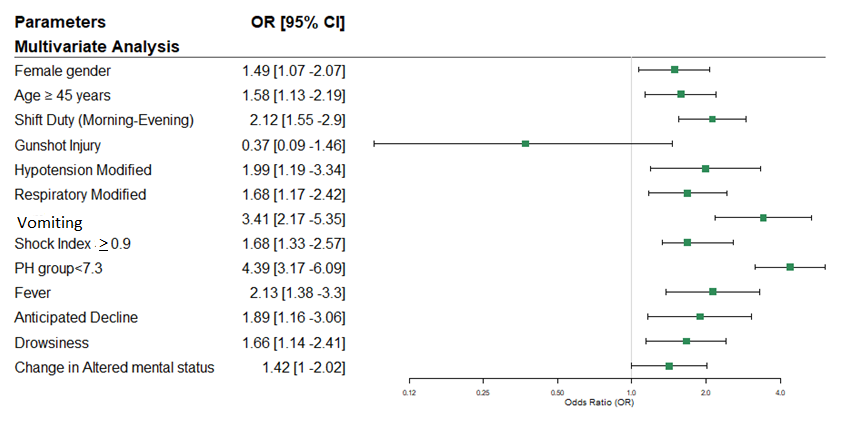


**Supplementary Figure 1: Multivariate Analysis of 12 variables for the Physiologically Difficult Airway Prediction Score**

**Supplementary Table 1: Univariate and multivariate binary logistic regression analysis to determine the predictors of physiological difficult airway during endotracheal intubation**

| Parameters | Univariate | | Multivariate – Initial level | | Multivariate – Final level | |
| --- | --- | --- | --- | --- | --- | --- |
|  | **OR [95% CI]** | **p-value** | **OR [95% CI]** | **p-value** | **OR [95% CI]** | **p-value** |
| Female gender | 1.91 [1.4 -2.6] | <0.001* | 1.48 [1.06 -2.07] | 0.022* | 1.49 [1.07 -2.07] | 0.017* |
| Age ≥ 45 years | 1.74 [1.28 -2.36] | <0.001* | 1.59 [1.13 -2.24] | 0.008* | 1.58 [1.13 -2.19] | 0.007* |
| Shift Duty (Morning-Evening) | 2.19 [1.64 -2.94] | <0.001* | 2.14 [1.56 -2.94] | <0.001* | 2.12 [1.55 -2.9] | <0.001* |
| Comma | 0.93 [0.44 -1.99] | 0.855 |  |  |  |  |
| Seizures | 0.63 [0.36 -1.09] | 0.098 | 1.3 [0.72 -2.36] | 0.383 |  |  |
| Trauma | 2.8 [1.7 -4.63] | <0.001* | 1.45 [0.76 -2.76] | 0.258 |  |  |
| Hypoexemia | 1.9 [1.4 -2.58] | <0.001* | 1.34 [0.94 -1.9] | 0.101 |  |  |
| Polytrauma | 0.53 [0.21 -1.35] | 0.185 | 1.83 [0.58 -5.7] | 0.3 |  |  |
| Isolated Trauma | 0.58 [0.25 -1.33] | 0.200 | 1.43 [0.59 -3.47] | 0.43 |  |  |
| Gunshot Injury | 0.36 [0.1 -1.27] | 0.112 | 0.42 [0.1 -1.66] | 0.214 | 0.37 [0.09 -1.46] | 0.156 |
| Ketamine | 1.9 [1.02 -3.51] | 0.042* | 0.94 [0.5 -1.76] | 0.844 |  |  |
| Propofol | 0.92 [0.67 -1.26] | 0.601 |  |  |  |  |
| Etomidate | 1.14 [0.81 -1.61] | 0.448 |  |  |  |  |
| Midazolam | 0.87 [0.65 -1.16] | 0.332 |  |  |  |  |
| Succinylcholine. | 0.64 [0.47 -0.87] | 0.005* | 0.73 [0.33 -1.62] | 0.436 | 0.67 [0.47 -0.97] | 0.032* |
| Rocuronium | 0.65 [0.39 -1.08] | 0.094 | 0.58 [0.23 -1.47] | 0.247 | 0.54 [0.29 -1] | 0.049* |
| Atracurium | 1.9 [1.35 -2.67] | <0.001* | 1.1 [0.49 -2.47] | 0.82 |  |  |
| Metabolic Acidosis | 1.86 [1.16 -2.99] | 0.01* | 1.01 [0.62 -1.65] | 0.965 |  |  |
| Hypotension Modified | 3.71 [2.34 -5.88] | <0.001* | 1.97 [1.17 -3.31] | 0.01* | 1.99 [1.19 -3.34] | 0.009* |
| Respiratory Modified | 2.62 [1.93 -3.56] | <0.001* | 1.7 [1.18 -2.45] | 0.005* | 1.68 [1.17 -2.42] | 0.005* |
| Vomiting | 3.09 [2.04 -4.67] | <0.001* | 3.51 [2.23 -5.53] | <0.001* | 3.41 [2.17 -5.35] | <0.001* |
| Shock Index > 0.9 | 2.17 [1.65 -2.99] | <0.001* | 1.36 [1.1 -1.95] | 0.048* | 1.68 [1.33 -2.57] | 0.032* |
| PH group < 7.3 | 3.61 [2.75 -4.73] | <0.001* | 4.44 [3.19 -6.17] | <0.001* | 4.39 [3.17 -6.09] | <0.001* |
| Fever | 2.53 [1.67 -3.82] | <0.001* | 2.13 [1.37 -3.31] | <0.001* | 2.13 [1.38 -3.3] | <0.001* |
| Anticipated Decline | 1.67 [1.08 -2.56] | 0.02* | 1.94 [1.19 -3.17] | 0.008* | 1.89 [1.16 -3.06] | 0.01* |
| GCS < 15 | 2.57 [1.85 -3.57] | <0.001* | 1.65 [1.13 -2.42] | 0.009* | 1.66 [1.14 -2.41] | 0.008* |
| Agitation | 1.36 [1 -1.84] | 0.048* | 1.42 [0.99 -2.05] | 0.059 | 1.42 [1 -2.02] | 0.048* |

**Supplementary Table 2: Training dataset threshold value of the score with sensitivity and specificity.**

| **Training Data Set /Treshold Value** | **Sensitivity [95% C.I]** | **Specificity [95% C.I]** | **PPV** | **NPV** | **LR+** | **TP** | **TN** | **FP** | **FN** | **Sensitivity+ Specificity** | **Accuracy** |
| --- | --- | --- | --- | --- | --- | --- | --- | --- | --- | --- | --- |
| 0 | 100% [95% C.I ; 99.1 -100] | 1.8% [95% C.I ; 0.6 -4.2] | 65.30% | 100.00% | 1.018 | 527 | 5 | 280 | 0 | 1.018 | 65.52% |
| 1 | 100% [95% C.I ; 99.1 -100] | 2.5% [95% C.I ; 1.1 -5.1] | 65.47% | 100.00% | 1.025 | 527 | 7 | 278 | 0 | 1.025 | 65.76% |
| 2 | 100% [95% C.I ; 99.1 -100] | 11.2% [95% C.I ; 8.1 -15.5] | 67.56% | 100.00% | 1.126 | 527 | 32 | 253 | 0 | 1.112 | 68.84% |
| 3 | 100% [95% C.I ; 99.1 -100] | 13% [95% C.I ; 9.6 -17.4] | 68.00% | 100.00% | 1.149 | 527 | 37 | 248 | 0 | 1.130 | 69.46% |
| 4 | 100% [95% C.I ; 99.1 -100] | 23.9% [95% C.I ; 19.3 -29.2] | 70.83% | 100.00% | 1.313 | 527 | 68 | 217 | 0 | 1.239 | 73.28% |
| 5 | 100% [95% C.I ; 99.1 -100] | 27.7% [95% C.I ; 22.8 -33.2] | 71.90% | 100.00% | 1.383 | 527 | 79 | 206 | 0 | 1.277 | 74.63% |
| 6 | 96.8% [95% C.I ; 94.8 -98] | 41.1% [95% C.I ; 35.5 -46.9] | 75.22% | 87.31% | 1.642 | 510 | 117 | 168 | 17 | 1.378 | 77.22% |
| 7 | 95.4% [95% C.I ; 93.3 -96.9] | 49.8% [95% C.I ; 44.1 -55.6] | 77.86% | 85.54% | 1.902 | 503 | 142 | 143 | 24 | 1.453 | 79.43% |
| 8 | 86% [95% C.I ; 82.7 -88.7] | 63.9% [95% C.I ; 58.1 -69.2] | 81.47% | 71.09% | 2.378 | 453 | 182 | 103 | 74 | 1.498 | 78.20% |
| **9** | 81.8% [95% C.I ; 78.2 -84.8] | 73% [95% C.I ; 67.5 -77.8] | **84.84%** | **68.42%** | **3.027** | **431** | **208** | **77** | **96** | **1.548** | **78.69%** |
| 10 | 67.9% [95% C.I ; 63.8 -71.8] | 80.4% [95% C.I ; 75.3 -84.5] | 86.47% | 57.54% | 3.457 | 358 | 229 | 56 | 169 | 1.483 | 72.29% |
| 11 | 60.5% [95% C.I ; 56.3 -64.6] | 86% [95% C.I ; 81.4 -89.5] | 88.86% | 54.08% | 4.313 | 319 | 245 | 40 | 208 | 1.465 | 69.46% |
| 12 | 47.4% [95% C.I ; 43.2 -51.7] | 92.3% [95% C.I ; 88.5 -94.9] | 91.91% | 48.70% | 6.145 | 250 | 263 | 22 | 277 | 1.397 | 63.18% |
| 13 | 39.3% [95% C.I ; 35.2 -43.5] | 96.5% [95% C.I ; 93.5 -98.1] | 95.39% | 46.22% | 11.194 | 207 | 275 | 10 | 320 | 1.358 | 59.36% |
| 14 | 29% [95% C.I ; 25.3 -33.1] | 98.2% [95% C.I ; 95.8 -99.4] | 96.84% | 42.81% | 16.548 | 153 | 280 | 5 | 374 | 1.273 | 53.33% |
| 15 | 21.1% [95% C.I ; 17.8 -24.8] | 99.6% [95% C.I ; 97.8 -100] | 99.11% | 40.57% | 60.028 | 111 | 284 | 1 | 416 | 1.207 | 48.65% |
| 16 | 12.3% [95% C.I ; 9.8 -15.4] | 100% [95% C.I ; 98.4 -100] | 100.00% | 38.15% | +Inf | 65 | 285 | 0 | 462 | 1.123 | 43.10% |
| 17 | 7.4% [95% C.I ; 5.5 -10] | 100% [95% C.I ; 98.4 -100] | 100.00% | 36.87% | +Inf | 39 | 285 | 0 | 488 | 1.074 | 39.90% |
| 18 | 4.7% [95% C.I ; 3.2 -7] | 100% [95% C.I ; 98.4 -100] | 100.00% | 36.21% | +Inf | 25 | 285 | 0 | 502 | 1.047 | 38.18% |
| 19 | 3% [95% C.I ; 1.9 -4.9] | 100% [95% C.I ; 98.4 -100] | 100.00% | 35.80% | +Inf | 16 | 285 | 0 | 511 | 1.030 | 37.07% |
| 20 | 1.5% [95% C.I ; 0.7 -3] | 100% [95% C.I ; 98.4 -100] | 100.00% | 35.45% | +Inf | 8 | 285 | 0 | 519 | 1.015 | 36.08% |
| 21 | 0.8% [95% C.I ; 0.2 -2] | 100% [95% C.I ; 98.4 -100] | 100.00% | 35.27% | +Inf | 4 | 285 | 0 | 523 | 1.008 | 35.59% |
| 22 | 0.2% [95% C.I ; 0 -1.2] | 100% [95% C.I ; 98.4 -100] | 100.00% | 35.14% | +Inf | 1 | 285 | 0 | 526 | 1.002 | 35.22% |
| 24 | 0% [95% C.I ; 0 -0.9] | 100% [95% C.I ; 98.4 -100] |  | 35.10% |  | 0 | 285 | 0 | 527 | 1.000 | 35.10% |
| ***Test is positive if Development Score > threshold value*** | | |  |  |  |  |  |  |  |  |  |

**Supplementary Table 3: Validation dataset threshold value of the score with sensitivity and specificity.**

| **Validation Data Set /Threshold Score** | **Sensitivity [95% C.I]** | **Specificity [95% C.I]** | **PPV** | **NPV** | **LR+** | **TP** | **TN** | **FP** | **FN** | **Sensitivity+ Specificity** | **Accuracy** |
| --- | --- | --- | --- | --- | --- | --- | --- | --- | --- | --- | --- |
| 0 | 100% [95% C.I ; 96.8 -100] | 4.5% [95% C.I ; 1.1 -13.2] | 69% | 100% | 1.048 | 143 | 3 | 63 | 0 | 1.045 | 69.9% |
| 1 | 100% [95% C.I ; 96.8 -100] | 7.6% [95% C.I ; 3 -17] | 70% | 100% | 1.082 | 143 | 5 | 61 | 0 | 1.076 | 70.8% |
| 2 | 100% [95% C.I ; 96.8 -100] | 13.6% [95% C.I ; 7.2 -24.2] | 72% | 100% | 1.158 | 143 | 9 | 57 | 0 | 1.136 | 72.7% |
| 3 | 100% [95% C.I ; 96.8 -100] | 15.2% [95% C.I ; 8.3 -26] | 72% | 100% | 1.179 | 143 | 10 | 56 | 0 | 1.152 | 73.2% |
| 4 | 100% [95% C.I ; 96.8 -100] | 37.9% [95% C.I ; 27.2 -50] | 78% | 100% | 1.610 | 143 | 25 | 41 | 0 | 1.379 | 80.4% |
| 5 | 100% [95% C.I ; 96.8 -100] | 42.4% [95% C.I ; 31.3 -54.5] | 79% | 100% | 1.737 | 143 | 28 | 38 | 0 | 1.424 | 81.8% |
| 6 | 95.1% [95% C.I ; 90 -97.8] | 50% [95% C.I ; 38.3 -61.7] | 80% | 83% | 1.902 | 136 | 33 | 33 | 7 | 1.451 | 80.9% |
| 7 | 90.2% [95% C.I ; 84.1 -94.2] | 59.1% [95% C.I ; 47 -70.1] | 83% | 74% | 2.205 | 129 | 39 | 27 | 14 | 1.493 | 80.4% |
| 8 | 81.1% [95% C.I ; 73.8 -86.7] | 74.2% [95% C.I ; 62.4 -83.3] | 87% | 64% | 3.149 | 116 | 49 | 17 | 27 | 1.554 | 78.9% |
| **9** | **74.1% [95% C.I ; 66.3 -80.6]** | **83.3% [95% C.I ; 72.3 -90.5]** | **91%** | **60%** | **4.448** | **106** | **55** | **11** | **37** | **1.575** | **77.0%** |
| 10 | 61.5% [95% C.I ; 53.3 -69.1] | 87.9% [95% C.I ; 77.5 -93.9] | 92% | 51% | 5.077 | 88 | 58 | 8 | 55 | 1.494 | 69.9% |
| 11 | 51.7% [95% C.I ; 43.6 -59.8] | 90.9% [95% C.I ; 81.1 -96] | 93% | 47% | 5.692 | 74 | 60 | 6 | 69 | 1.427 | 64.1% |
| 12 | 36.4% [95% C.I ; 28.9 -44.5] | 95.5% [95% C.I ; 86.8 -98.9] | 95% | 41% | 8.000 | 52 | 63 | 3 | 91 | 1.318 | 55.0% |
| 13 | 28% [95% C.I ; 21.3 -35.9] | 97% [95% C.I ; 88.8 -99.7] | 95% | 38% | 9.231 | 40 | 64 | 2 | 103 | 1.249 | 49.8% |
| 14 | 18.9% [95% C.I ; 13.3 -26.2] | 97% [95% C.I ; 88.8 -99.7] | 93% | 36% | 6.231 | 27 | 64 | 2 | 116 | 1.159 | 43.5% |
| 15 | 14.7% [95% C.I ; 9.8 -21.5] | 97% [95% C.I ; 88.8 -99.7] | 91% | 34% | 4.846 | 21 | 64 | 2 | 122 | 1.117 | 40.7% |
| 16 | 7.7% [95% C.I ; 4.3 -13.4] | 97% [95% C.I ; 88.8 -99.7] | 85% | 33% | 2.538 | 11 | 64 | 2 | 132 | 1.047 | 35.9% |
| 17 | 6.3% [95% C.I ; 3.2 -11.7] | 98.5% [95% C.I ; 91 -100] | 90% | 33% | 4.154 | 9 | 65 | 1 | 134 | 1.048 | 35.4% |
| 18 | 4.2% [95% C.I ; 1.8 -9.1] | 98.5% [95% C.I ; 91 -100] | 86% | 32% | 2.769 | 6 | 65 | 1 | 137 | 1.027 | 34.0% |
| 19 | 2.1% [95% C.I ; 0.5 -6.3] | 98.5% [95% C.I ; 91 -100] | 75% | 32% | 1.385 | 3 | 65 | 1 | 140 | 1.006 | 32.5% |
| 20 | 0.7% [95% C.I ; 0 -4.3] | 98.5% [95% C.I ; 91 -100] | 50% | 31% | 0.462 | 1 | 65 | 1 | 142 | 0.992 | 31.6% |
| 21 | 0.7% [95% C.I ; 0 -4.3] | 100% [95% C.I ; 93.2 -100] | 100% | 32% | +Inf | 1 | 66 | 0 | 142 | 1.007 | 32.1% |
| 22 | 0% [95% C.I ; 0 -3.2] | 100% [95% C.I ; 93.2 -100] |  | 32% |  | 0 | 66 | 0 | 143 | 1.000 | 31.6% |
